# Content of Zinc and Copper in Selected Plants Growing Along a Motorway

**DOI:** 10.1007/s00128-015-1648-8

**Published:** 2015-09-12

**Authors:** Elżbieta Malinowska, Kazimierz Jankowski, Beata Wiśniewska-Kadżajan, Jacek Sosnowski, Roman Kolczarek, Jolanta Jankowska, Grażyna A. Ciepiela

**Affiliations:** Department of Grassland and Landscape Architecture, Siedlce University of Natural Sciences and Humanities, B. Prusa 14 Street, 08-110 Siedlce, Poland; Laboratory of Agrometeorology and Fundamentals of Land Reclamation, Siedlce University of Natural Sciences and Humanities, Siedlce, Poland; Department of Tourism and Recreation, Siedlce University of Natural Sciences and Humanities, Siedlce, Poland

**Keywords:** Selected heavy metals, Plants, Roadside, Poland

## Abstract

In 2011 a study was carried out analyzing the effects of road traffic on bioaccumulation of zinc and copper in selected species of dicotyledonous plants growing on adjacent grasslands. To do the research the plants were sampled from the 9-km-long Siedlce bypass, a part of the international route E-30. They were collected during the flowering stage, at following distances from the road: 1, 5, 10, 15 m. The content of zinc and copper was determined with the AAS method, with dry mineralisation done before. The highest concentration of the elements, regardless of the distance from the road, was found in *Taraxacum* spec. Among the tested plants, the lowest zinc content was in *Vicia cracca*, and the lowest copper content in *Rumex acetosa*. The limit for copper content was exceeded in *Taraxacum* spec. and, slightly, in *Achillea millefolium* growing at the roadside, closest to the roadway.

Car traffic is a considerable source of heavy metals (Diatta et al. 2003; Swaileh et al. 2004; Jozic et al. 2009; Jankowski et al. 2015), which are believed to be very dangerous roadside pollutants (Viard et al. 2004; Petrotou et al. 2012). In addition to lead and cadmium, believed to be an inseparable part of dust issued by motor vehicles, zinc and copper are among the most common soil and vegetation pollutants in the area of communication routes. Excessive concentration of heavy metals in soil (Pb, Zn, Cr, Co and Cu) may decrease the number of leaves and the rate of shoot growth.

It may cause chlorosis, leaf necrosis, discoloration and shortage in macro and micronutrients (Pandey and Sharma 2002; Rout and Das 2003; Rao 2006; Doğanlar and Atmaca 2011; Kandziora-Ciupa et al. 2013). Some important causes of elevated heavy metal content in roadside areas are: exhaust fumes of motor vehicles, abrasion of tyres and road surfaces, wear of brake linings, wear of moving engine parts, corrosion of various vehicle components, as well as oil spills.

Significant quantities of zinc and copper come from soot and heavy metal oxides, used as tyre manufacturing additives, which, flowing off the road, enter the soil after precipitation. This kind of pollution clearly contributes to an increase of zinc, copper, as well as lead content in the environment. According to many publications (Jankowski et al. 2015) the amount of heavy metals is increased near main roads.

Thus, in those areas there is a need for systematic and detailed research on the accumulation of heavy metals in the soil-crop system (Van Bohemen and Van de Laak 2003).

The aim of the research is to assess, in terms of fodder suitability, the relation between the distance of the plants from the motorway and zinc and copper bioaccumulation in selected dicotyledonous species growing along the road on meadows and pastures.

## Materials and Methods

The plant samples for the research were above-ground parts of four different plant species: *Taraxacum* spec*., Achillea millefolium* L.*, Rumex acetosa* L. and *Vicia cracca* L. growing on grasslands adjacent to the road. The selection of species was determined by their different types of leaf lamina and inflorescence, which have an impact on dust retention. Among the analyzed species, *V. cracca* has soft hairy stems and leaves, while *A. millefolium* grows bushy branches with pinnate leaves and blooms the longest, from May until autumn. *R. acetosa* and *Taraxacum* spec. produce a lot of leaves of large surface. The plants for the study were sampled from along the international route E-30, the Siedlce bypass, in May 2011, with a 9 km stretch serving for the research. Samples, with the total number of 96, were taken in three replicates on both sides, with the following distances from the edge of the road: 1, 5, 10 and 15 m. Then, from the bulk samples, 5 samples of each plant species from every distance from the road were selected. The plant material sampled in such a manner was dried at 105°C, until dry matter was obtained. Then 1 g of the material was put to a porcelain pot and dry-mineralized at 450°C for 18 h and then solved in 10 % HNO_3_. To determine heavy metals content soil samples were taken in the same places as plant samples. Zinc and copper content in the plants and in the soil was determined with the AAS methods using the spectrophotometer Varian Spectra AA-20 (Varian, Australia).

The national road 2 is a part of the international route E-30 from Cork, Ireland, to Omsk in Russia. The test area, about 80 km east of Warsaw, is part of the Masovian Province, which is located in central-eastern Poland. In 2010 three measurements of daily traffic of motor vehicles on Polish roads were taken by the General Directorate for National Roads and Motorways. The average daily motor vehicle traffic for the network of national roads in 2010 was 9888 vehicles per day. On international roads, the average daily motor vehicle traffic was 16,667 vehicles per day, while on other national roads the traffic was 7097 vehicles per day. Along the analyzed stretch, the Siedlce bypass, the average daily movement of motor vehicles was higher than the average volume of traffic on Polish national roads and amounted to 8136 vehicles per day (General Directorate for National Roads and Motorways 2010).

In the experiment arithmetic means, coefficients of variation (CV) and geo-accumulation index (*I*_geo_) were calculated. The index of geo-accumulation (*I*_geo_) enables the assessment of contamination by comparing the current and the pre-industrial concentrations of the metals in earth crust (Loska et al. 1997; Iqbal and Shah 2011). It is calculated using the following mathematical formula: *I*_geo_ = log*C*_*n*_/1.5*B*_*n*_ (*C*_*n*_—concentration of the element in the given aspect of environment; *B*_*n*_—geochemical basic level for the element). The factor 1.5 is introduced to minimize the effect of possible variations in the background values which may be attributed to lithogenic variations. *I*_geo_ values were interpreted as; *I*_geo_ ≤ 0—practically uncontaminated, 0 < *I*_geo_ < 1—uncontaminated to moderately contaminated, 1 < *I*_geo_ < 2—moderately contaminated, 2 < *I*_geo_ < 3—moderately to heavily contaminated, 3 < *I*_geo_ < 4—heavily contaminated, 4 < *I*_geo_ < 5—heavily to extremely contaminated and 5 > *I*_geo_—extremely contaminated.

The results of the research were processed statistically using Statistica Version 10.0, StatSoft. The impact of the factors influencing geo-accumulation of zinc and copper was determined using two-way analysis of variance. For detailed comparison of means, Tukey’s test was used at *p* ≤ 0.05. The value of correlation coefficient at *p* ≤ 0.05 was worked out to determine the relationship between the content of zinc and copper in the plants and in the soil.

## Results and Discussion

In the research, zinc and copper content in the sample plants was measured to assess whether those plants could be used as livestock feed. For this purpose the concentration of those elements was compared to the maximum limits set by the Minister of Agriculture and Rural Development in the Regulation of 23 January 2007 on the permissible content of undesirable substances in fodder (Regulations of the Minister for Agriculture and Rural Development 2007), with the limit value of zinc concentration ranging from 50 to 100 mg of Zn kg^−1^. According to Kabata-Pendias et al. (1993) maximum limits of heavy metals content in fodder are as follows: <100 mg Zn, <30 mg Cu, <20 mg Cr, <50 mg Ni, <10 mg Pb, and <0.5 mg Cd, all in mg kg^−1^ D.M. Biomass with a higher content of heavy metals should not be used as livestock feed but for compost or combustion.

The content of zinc, regardless of the distance from the road, in the selected dicotyledonous plants showed significant diversities, depending on the analyzed plant species (Table 1). The highest accumulation of the element was observed in *Taraxacum* spec. (63.24 mg kg^−1^ D.M.), the lowest (46.08 mg kg^−1^ D.M.) in *V. cracca*. Concentration of heavy metals in various species of the genus *Taraxacum* is considered to indicate the level of bioaccumulation of heavy metals in the environment (Kabata-Pendias and Dudka 1991). The average zinc concentration in the tested species was 55.43 mg kg^−1^ of dry matter.

**Table 1:** The content of zinc and copper in plant samples (mg kg^−1^ D.M.)

Plants species	Zinc	Copper
*Taraxacum* spec.	63.24	14.37
*Achillea millefolium*	58.35	10.76
*Rumex acetosa*	54.06	7.55
*Vicia cracca*	46.08	8.17
Average	55.43	10.21
LSD_0.05_	6.60	1.74

Confidence interval *p* ≤ 0.05

In Poland, concentration of zinc in plants ranges from 10 to 50 mg kg^−1^ D.M., depending on the plant species (Kabata-Pendias 2004) while Kalny et al. (2007) say that zinc concentration in dry matter of *Taraxacum* is 19.1 mg kg^−1^.

In the research of Królak (2001), carried out in southern Podlasie, the average content of zinc in leaves of *Taraxacum* spec. was 65.8 mg kg^−1^, while in roots it was 41.4 mg kg^−1^.

The chemical analysis of plants showed significant differences in the content of zinc, depending on the species of a plant and distance from the road (Table 2). Among the analyzed plants, the highest accumulation was observed in *R. acetosa* (67.23 mg kg^−1^) collected 1 m away from the roadway, then in *Taraxacum* spec., 5 and 10 m away (91.27 and 81.41 mg kg^−1^, respectively) and in *A. millefolium* (80.65 mg kg^−1^), 5 m away from the road. The highest content of zinc, the average of the species, was observed in the plants growing 5 m away from the road. The average content of zinc in the test plants (regardless of the species) decreases with the increase in distance from the road, except for the plants gathered from the roadside, 5 m away from the road. The content of zinc in dry matter of the plants never exceeded 100 mg kg^−1^, which allows using them as fodder. According to Polechonska et al. (2013) *Polygonum aviculare* has a higher accumulation of Cd, Cu, Fe, Ni, Pb and Zn caused by traffic. Plants with an established tendency to bio-accumulate heavy metals in leaves or roots are used in biological monitoring. One of such species is *Taraxacum officinale*, in which considerable amounts of chemical pollutants accumulate (Keane et al. 2001). It is a perennial plant growing new leaves every year, which helps to distinguish between air pollutants and soil pollutants. The fact that it commonly grows in nearly all habitats decides that it is used as photo indicator of environmental pollution (Kuleff and Djingova 1984; Simon et al. 1996).

**Table 2:** The content of zinc and copper in plant samples in the studied distances (mg kg^−1^ D.M.)

Distance from the road (m)	Plant species	Zinc		Copper	
		Average	Coefficient of variation (%)	Average	Coefficient of variation (%)
1 m	*Taraxacum* spec.	55.73	22.23	21.36	14.45
	*Achillea millefolium* L.	59.57	16.72	12.46	9.33
	*Rumex acetosa* L.	67.23	20.45	9.58	11.48
	*Vicia cracca* L	46.32	24.87	10.89	13.40
Average for 1 m		57.21	21.07	13.56	12.17
5 m	*Taraxacum* spec.	91.27	10.34	11.99	13.51
	*Achillea millefolium* L.	80.65	9.27	10.25	12.21
	*Rumex acetosa* L.	62.78	14.45	7.69	13.08
	*Vicia cracca* L.	59.08	28.35	8.35	18.05
Average for 5 m		73.45	15.60	9.57	14.21
10 m	*Taraxacum* spec.	81.41	27.22	11.07	20.01
	*Achillea millefolium* L.	47.61	38.48	8.26	19.04
	*Rumex acetosa* L.	50.45	39.88	6.89	11.10
	*Vicia cracca* L.	40.12	31.79	7.75	9.59
Average for 10 m		54.90	34.34	8.49	14.94
15 m	*Taraxacum* spec.	24.56	54.85	15.89	25.64
	*Achillea millefolium* L.	44.91	23.15	9.24	20.85
	*Rumex acetosa* L.	35.78	30.29	5.74	16.51
	*Vicia cracca* L.	38.78	26.41	6.05	17.25
Average for 15 m		36.17	33.68	9.23	20.06
LSD_0.05_ for: A—distance		A = 2.47		A = 0.937	
B—plants species		B = 2.47		B = 0.937	
A × B—interaction		A × B = 4.94		A × B = 1.87	
B × A—interaction		B × A = 4.94		B × A = 1.87	

Confidence interval *p* ≤ 0.05

Depending on the species, accumulation of copper is highly diversified (Table 1). The highest content of this element, the average from all locations, was noted for *Taraxacum* spec. (14.37 mg kg^−1^), the lowest for *R. acetosa* (7.55 mg kg^−1^) and *V. cracca* (8.17 mg kg^−1^).

Based on the findings, it was established that heavy metal content was substantially higher in the locations closest (1 m) to the highway (Table 2). In all the plants growing closest to the road, except for *R. acetosa*, the limits for copper in fodder were exceeded and in the case of *Taraxacum* spec. more than doubled. It was observed that the content of copper decreased with the growing distance from the road in two species: *R. acetosa* and *V. cracca*. According to Djingova and Kuleff (1993), the leaves of *Taraxacum* spec. contain 13.8 mg kg^−1^. The average concentration of copper in *Tanacetum vulgare* growing in the area of the Bełchatow Lignite Mine was more or less the same as the concentration in plants growing along main roads (Jasion et al. 2013). According to framework guidelines for agriculture (1993), maximum limit for copper is 25 mg kg^−1^.

The lowest copper content was found in *R. acetosa*, from 5.74 to 9.58 mg kg^−1^, and in *V. cracca*, from 6.05 to 10.89 mg kg^−1^. The highest values were reported for *Taraxacum* spec., from 11.07 to 21.36 mg kg^−1^, which excludes this plant from fodder use. The content of copper in *A. millefolium* ranged from 12.46 to 8.26 mg kg^−1^. In the case of this plant, the limits in the closes roadside were slightly exceeded.

**Fig. 1:**
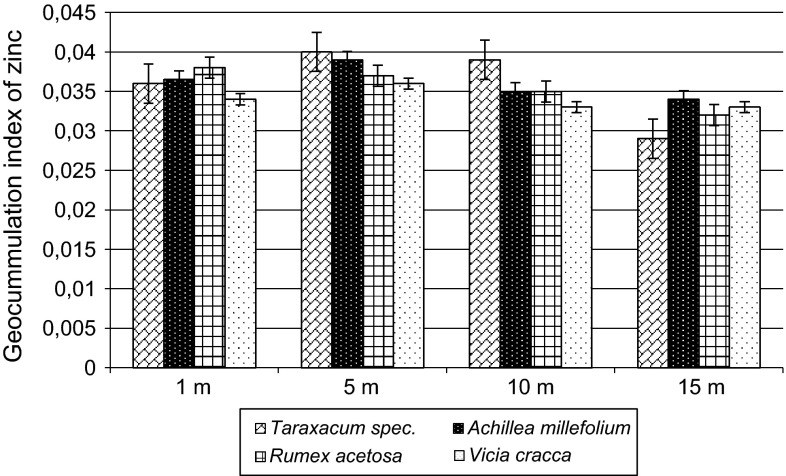
Value of geo-accumulation index (*I*_geo_) for average content of zinc in the selected plants

The value of the geo-accumulation index (*I*_geo_) (Fig. 1) for average content of zinc in the test species from each distance from the roadway ranges from 0.029 to 0.039. The reference value applied was zinc content in the soil of Poland, 32.4 mg kg^−1^ (Terelak et al. 2000). According to Loska et al. (2004), the obtained values indicate that the plants belong to class 1 of geo-accumulation index (0 < *I*_geo_ < 1), i.e. they are lightly polluted. Geo-accumulation index had widely been used in trace metal studies of sediments and soils (Amin et al. 2009; Singh et al. 2005).

**Fig. 2:**
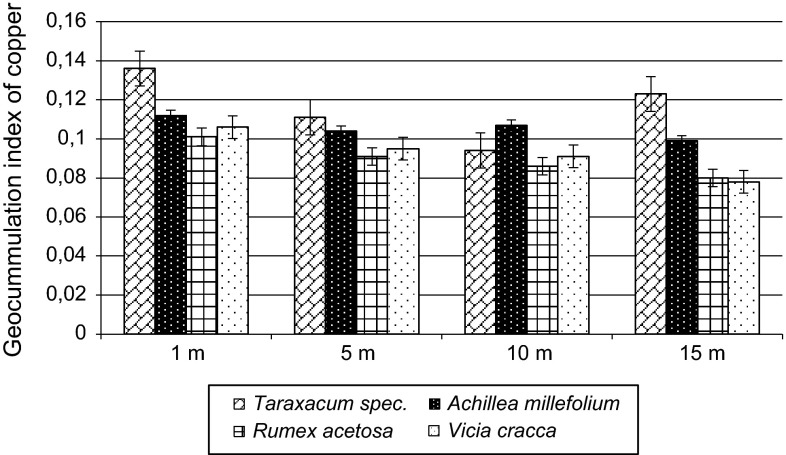
Value of geo-accumulation index (*I*_geo_) for average content of copper in the selected plants

The value of the geo-accumulation index (*I*_geo_) (Fig. 2) for average content of copper in the test species from each distance from the roadway ranges from 0.078 to 0.136. The average concentration of copper in soil in Poland, according to Terelak et al. (2000), is 6.50 mg kg^−1^.

Based on the calculated values, it can be said that the concentration of copper in sample plants indicates slight pollution.

The geo-accumulation index allows classification of how environmental elements are polluted by an element. It helps to assess if plants growing on the roadside can be used as animal fodder.

There was a considerable diversity of zinc and copper content in the soil sampled at different distances from the road (Table 3). The highest content of those elements was found in soil taken 5 m and 1 m from the highway, the lowest in places most distant from the traffic. The experiment showed that zinc and copper content in the soil samples was much lower than average normal content of those elements in Polish soils, which is commonly used as a comparative value (Terelak et al. 2000). There was a considerable relationship between the plant species and the zinc content in the soil where the plant grew, but not in the case of copper.

**Table 3:** The content of zinc and copper in soil samples in the studied distances (mg kg^−1^ D.M.)

Distance from the road (m)	Soil taken from the place of the plant’s growth-point	Zinc	Copper
1 m	*Taraxacum* spec.	20.35	4.23
	*Achillea millefolium* L.	18.69	3.28
	*Rumex acetosa* L.	17.21	3.69
	*Vicia cracca* L	20.01	4.01
Average for 1 m		19.07	3.80
5 m	*Taraxacum* spec.	30.12	4.20
	*Achillea millefolium* L.	28.52	4.65
	*Rumex acetosa* L.	26.12	3.56
	*Vicia cracca* L.	29.01	3.74
Average for 5 m		28.44	4.04
10 m	*Taraxacum* spec.	18.23	2.36
	*Achillea millefolium* L.	15.42	2.47
	*Rumex acetosa* L.	17.41	3.01
	*Vicia cracca* L.	16.51	2.89
Average for 10 m		16.89	2.68
15 m	*Taraxacum* spec.	14.23	2.47
	*Achillea millefolium* L.	15.69	2.58
	*Rumex acetosa* L.	13.02	2.69
	*Vicia cracca* L.	16.84	2.55
Average for 15 m		14.95	2.57
LSD_0.05_ for: A—distance		A = 0.616	A = 0.428
B—plants species		B = 0.616	B = n.s.
A × B—interaction		A × B = 1.23	A × B = 0.855
B × A—interaction		B × A = 1.23	B × A = 0.855

*n.s.* Not significant difference, confidence interval *p* ≤ 0.05

Correlation coefficient between zinc and copper content in the soil and in the plants did not show significant relationship. Thus, it can be said that accumulation of heavy metals by the plants tested was caused mainly by dust emitted by road traffic and falling on those plants. Among the analyzed species the highest concentration of zinc and copper, regardless of the distance from the road, was found in *Taraxacum* spec., the lowest zinc content was in *V. cracca*, and the lowest copper content was in *R. acetosa.*

There were significant differences in the concentration of heavy metals, depending on the species of a plant and the distance from the roadway. The biggest amounts of zinc were accumulated by the plants growing 5 m away from the road, while for copper just 1 m away.

The zinc concentration in the test plant material was lower than the proposed threshold amounts in plants intended for fodder, while the concentration of copper significantly exceeded the standards in *Taraxacum* spec., regardless of the collection point and in *A. millefolium* collected directly at the highway.

It was found that the most appropriate plant for biomonitoring in grasslands is *Taraxacum* spec. because it is characterized by the highest bioaccumulation of the examined elements.

Zinc and copper content in the soil sampled at different distances from the road was much lower than the average content of those elements in Polish soils, which is commonly used as a comparative value. Considering this it can be said that heavy metals accumulation in tested plants was caused mainly by falling dust coming from road traffic.
